# High-Quality Draft Genome Sequence of the Type Strain of Allorhizobium vitis, the Primary Causal Agent of Grapevine Crown Gall

**DOI:** 10.1128/MRA.01045-18

**Published:** 2018-09-06

**Authors:** Han Ming Gan, Melvin Vun Jye Lee, Michael A. Savka

**Affiliations:** a Centre for Integrative Ecology, School of Life and Environmental Sciences, Deakin University, Geelong, Victoria, Australia; b Deakin Genomics Centre, Deakin University, Geelong, Victoria, Australia; c School of Science, Monash University Malaysia, Bandar Sunway, Malaysia; d The Gosnell School of Life Sciences, Rochester Institute of Technology, Rochester, New York, USA; University of Arizona

## Abstract

Using Illumina and Nanopore reads, we assembled a high-quality draft genome sequence of Allorhizobium vitis K309^T^ (= ATCC 49767^T^, = NCPPB 3554^T^), a phytopathogenic strain isolated from a grapevine in Australia. The hybrid approach generated 50% fewer contigs and a 3-fold increase in the *N*_50_ value compared with the previous Illumina-only assembly.

## ANNOUNCEMENT

Crown gall disease (CGD) of grapevine is a chronic disease that occurs in vineyards worldwide (1). The causal agent of CGD is commonly referred to as Agrobacterium vitis, which was recently reclassified to the genus Allorhizobium based on whole-genome phylogeny (2, 3). Virulent strains harbor a tumor-inducing (Ti) plasmid that encodes functions that cause unregulated plant cell enlargement and division that leads to the appearance of CGD tumors that synthesize novel compounds known as opines (1, 4, 5). The type strain of Allorhizobium vitis, known as K309, was isolated in 1977 from grapevine in southern Australia. The resulting K309 galls contain octopine, and this strain catabolizes octopine as a sole carbon and nitrogen source (6). The complete genome sequence of *A. vitis* strain S4 is the only *A. vitis* genome sequence that has been published to date (7). To further contribute to the genomic resource for this species, we report the high-quality whole-genome sequence of its type strain, Allorhizobium vitis K309.

Approximately 10 bacterial colonies were scraped from a 3-day-old potato dextrose agar culture using a sterile 200-µl pipette tip and transferred into SDS lysis buffer (8). Genomic DNA purification was subsequently performed as previously described (8). For Illumina sequencing, DNA was processed with the Nextera XT library preparation kit (Illumina, San Diego, CA, USA) and sequenced on the MiSeq desktop sequencer (2 × 250-bp run configuration). A total of 1 µg of DNA was processed and sequenced using the SQK-MAP-104 kit (Oxford Nanopore, UK) and R9 chemistry, respectively, as previously described (9). Basecalling of the nanopore reads was performed with Albacore v2.3.1 (Oxford Nanopore). Adapter trimming of the Illumina reads was performed using Trimmomatic v0.3.6 (10), and Nanopore reads shorter than 1,000 bp were removed. The processed reads were assembled with Unicycler v0.4.4 (11).

Hybrid assembly of 1.5 million Illumina paired-end reads and 5,224 Nanopore reads generated 22 contigs with a total length of 5.75 megabases (*N*_50_ value, 999,201 bp; GC content, 57.55%), presenting a substantial improvement to the unpublished first draft genome sequence of strain K309^T^ (GenBank accession number LMVL01000000; 42 contigs; *N*_50_ value, 331,122 bp). The NCBI Prokaryotic Genome Annotation Pipeline (PGAP) (12) predicted 4,998 protein-coding sequences, 3 rRNAs, and 45 tRNAs. A similarity search using the A. vitis Ti plasmid *virC* gene fragment (GenBank accession number AB465459) as the BLASTN query sequence identified contig8 (∼200 kb) as the putative Ti plasmid of A. vitis strain K309^T^. The genome size of strain K309 is 500 kb smaller than that of strain S4, with a pairwise average nucleotide identity (ANI) of less than 95% (92.81%) (13). The low pairwise ANI value suggests that strain S4 may represent a different genomospecies than A. vitis given the type strain status of strain K309, thus warranting future taxonomic investigation (14–16).

### Data availability.

This whole-genome shotgun project has been deposited at DDBJ/ENA/GenBank under the accession number LMVL00000000. The version described in this paper is the second version, LMVL02000000 (BioProject number PRJNA300487; BioSample number SAMN04223557). Illumina reads are available under SRA accession number SRP154038, and Nanopore basecalled fasta reads have been deposited at the Zenodo database (https://doi.org/10.5281/zenodo.1315327).
